# A Coarse-Grained Molecular Dynamics Description of Docetaxel-Conjugate Release from PLGA Matrices

**DOI:** 10.1021/acs.biomac.2c00903

**Published:** 2022-10-14

**Authors:** Martina Pannuzzo, Alessia Felici, Paolo Decuzzi

**Affiliations:** Laboratory of Nanotechnology for Precision Medicine, Fondazione Istituto Italiano di Tecnologia, Via Morego 30, Genoa16163, Italy

## Abstract

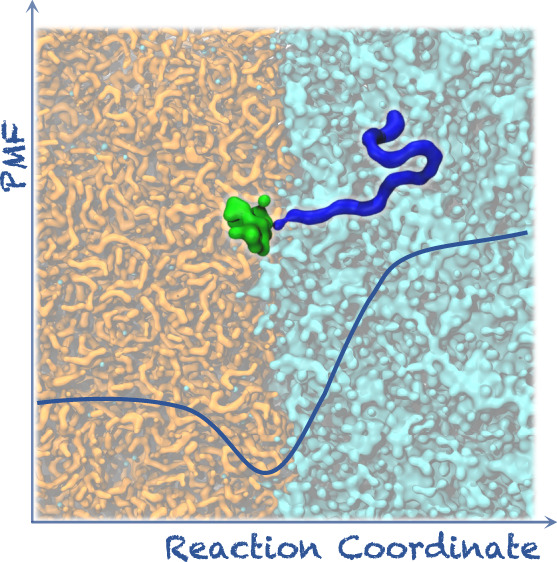

Despite the extensive use of poly-lactic-glycolic-acid (PLGA) in biomedical applications, computational research on the mesoscopic characterization of PLGA-based delivery systems is limited. In this study, a computational model for PLGA is proposed, developed, and validated for the reproducibility of transport properties that can influence drug release, the rate of which remains difficult to control. For computational efficiency, coarse-grained (CG) models of the molecular components under consideration were built using the MARTINI force field version 2.2. The translocation free energy barrier Δ*G*_t_^*^ across the PLGA matrix in the aqueous phase of docetaxel and derivatives of varying sizes and solubilities was predicted via molecular dynamics (MD) simulations and compared with experimental release data. The thermodynamic quantity Δ*G*_t_^*^ anticipates and can help explain the release kinetics of hydrophobic compounds from the PLGA matrix, albeit within the limit of a drug concentration below a critical aggregation concentration. The proposed computational framework would allow one to predict the pharmacological behavior of polymeric implants loaded with a variety of payloads under different conditions, limiting the experimental workload and associated costs.

## 1 Introduction

In recent decades, polymer-based delivery platforms have attracted increasing attention due to the ability to release therapeutics at a controlled rate.^1^ The release profile can be customized in terms of quantity of drug released and release time depending on the therapeutic application. However, experimental optimization of drug release kinetics requires intensive, time-consuming, and costly optimization steps. Molecular dynamics simulations can be used as a predictive tool to reduce the experimental workload. Coarse-grained (CG) models and enhanced sampling approaches can be combined to describe or phenomenologically explain drug release kinetics that occur at large spatial and temporal scales not easily accessible by atomistic simulations and unbiased approaches. Among polymer candidates, the FDA-approved poly-lactic-glycolic-acid (PLGA) is a biodegradable and safe polymer largely used for the manufacture of delivery systems. It has been used to release small molecules and biologicals mostly against cancer and cardiovascular diseases and in implantable devices, for the long-term and sustained release of therapeutic molecules to modulate inflammation and tissue regeneration.^2^

Typically, drug release kinetics will depend on drug-related parameters such as solubility, size, and shape as well as carrier-related factors such as composition, shape, size, and stiffness—the higher the drug retention within the carrier, the slower the drug release rate. The prodrug design, in which the drug is covalently conjugated to a moiety, has proven to be a valuable strategy for adjusting the solubility, shape, and size of the therapeutic agent toward improving the drug retention. Furthermore, when exposed to the particle surface, the conjugated moiety can bind a receptor and thus mediate the anchoring of the drug or particle to a biological target. Also, in response to environmental stimuli, it can become susceptible to cleavage so as to facilitate the release of the drug in its active configuration in a desired site.^3^

The impact of the mechanical and physicochemical properties of PLGA-based materials on drug release kinetics has been extensively studied and characterized experimentally. After fabrication, the drug trapped in the amorphous PLGA matrix is poorly mobile. Upon suspension of the particle in an aqueous solvent, water permeates the particle and acts as a plasticizer. As a consequence, the mechanical and physicochemical properties of the polymer matrix will change, as will do the transport properties. The PLGA polymer begins to degrade due to the hydrolytic cleavage of its polyester backbone, becoming more and more hydrophilic as it shortens. In this phase, the translocation of the drug from the polymer matrix into the aqueous solvent intertwines with the simultaneous efflux of polymer fragments. Despite the extensive use of PLGA in biomedical applications, in silico investigations remain limited due to the lack of a PLGA model suitable to investigate large-scale phenomena.^4a,4b^ We have recently developed and validated an atomistic model for PLGA relying on the 2016H66 force field to investigate the physicochemical and mechanical properties of a pure PLGA matrix and PLGA/polyethylene glycol (PEG) mixtures.^5^

In this study, molecular models of PLGA, free docetaxel (DTXL), and DTXL conjugated to moieties of varying solubilities and sizes are constructed at CG resolution for computational efficiency, employing the MARTINI force field (version 2.2) as to preserve chemical diversity.^6^ The CG PLGA model is validated for the reproducibility of the polymer matrix properties computed at atomistic resolution^5^ relevant for the release of encapsulated compounds into the aqueous solution. Finally, the polymer–water partition of the compounds predicted via simulations^7−9^ is discussed in relation to their experimentally determined release kinetics. Here, the focus is on the translocation of the drug entrapped within an intact polymer matrix (from 0 up to 3–7 days), prior to erosion and degradation.^10^

## 2 Methods

### 2.1 Molecular Dynamics Simulations

#### 2.1.1 Models

The chemotherapeutic molecule DTXL was considered as a test drug,^9^ both in its free form or conjugated to other molecules, like oleic acid (OA) and PEG chains with two different degrees of polymerization *N* (*N* = 10 and 25). Atomistic topologies for PLGA, DTXL, PEG_10_ (PEG chain with *N* = 10) and PEG_25_ (PEG chain with *N* = 25), water (W), octanol (O), and acetone (PPN) solvents were built using the united-atom (UA) 2016H66 force field.^11^ CG topologies for PLGA, DTXL, DTXL–oleic acid conjugate (OA–DTXL), and PEG–DTXL conjugates with PEG chains of two different lengths (PEG_10_–DTXL and PEG_25_–DTXL) were constructed according to the definition of the MARTINI forcefield v2.2.^6^ CG models for OA and PEG moieties, ions, W, O, and PPN solvents were readily available from the MARTINI website (http://cgmartini.nl). Following a similar approach as for the PEG parametrization,^12^ the PLGA and DTXL models were built aiming at reproducing partitioning free energies computed at atomistic resolution for the selected solvents.

#### 2.1.2 Alchemical Calculations

The alchemical free energy method^7^ was used to determine the solubility of the following species in selected solvents at the atomistic and CG levels: (1) a dimeric PLGA unit in PPN, O, and W solvents; (2) DTXL in O and W solvents; and (3) PEG chains with degrees of polymerization *N* = 10 and 20 in a polymer matrix consisting of PLGA chains with *N* = 10 and in W. In alchemical simulations, the solute explores a series of non-physical intermediates generated by progressively turning off the interactions with the solvent using a coupling parameter lambda λ (λ = 0, 0.1,..., 1). For atomistic models with partial atomic charges, Coulomb and Lennard–Jones components of the λ vector were turned off sequentially (20 λ windows in total), whereas for the uncharged CG systems, only the Lennard–Jones potential was considered (10 λ windows in total). Soft-core (sc) potentials were selected to smooth interactions at the cutoff distance to avoid large fluctuations deriving from the superposition of particles when the interaction energy is weak (near the end points λ = 0 and λ = 1). Each window was sampled for 20 ns at 300 K using the stochastic dynamics (SD) integrator. The multistate Bennett acceptance ratio (MBAR)^13^ method was employed to estimate the solvation free energy differences between λ = 0 and λ = 1 and statistical errors.

#### 2.1.3 PLGA–W Partition

The free energy translocation of the drugs (DTXL) and prodrugs (DTXL–OA, PEG_10_–DTXL, PEG_25_–DTXL) from an equilibrated PLGA matrix to the aqueous solution (the reaction coordinate representing the release progress) was computed at the CG level by the umbrella sampling (US) approach.^8^

The PLGA matrix consisted of chains with degree of polymerization *N* = 10 to ensure reasonable mobility and relaxation time. The US method allows to improve the sampling of rare events (e.g., a hydrophobic compound visiting the W phase) through the use of a bias potential that forces the compound of interest to explore regions that would otherwise not be energetically accessible. The unbiased free energy profile can be recovered by subtracting the known bias potential from the biased probability distribution obtained from simulations.

As for the US protocol, the COM of the drug or prodrug molecules was pulled away, once for each drug–polymer system, from the COM of the PLGA matrix toward the aqueous solution (along the z direction, perpendicular to the W–polymer interface) and starting structures for the umbrella simulations were extracted at 0.2 nm intervals for a total of 60 adjacent windows (see Figure S5 for sample starting configurations). Preliminary testing helped refine (1) the force constant to ensure proper overlapping of neighbor windows along the reaction coordinate, (2) the box dimensions to avoid artifacts deriving from periodic boundary conditions (PBC), and (3) the simulation time to reduce statistical uncertainties. Before the production run, each starting configuration was equilibrated for 100 ns with constraints applied to the compound embedded within a PLGA matrix. Each window was next sampled for 1 μs, with the distance between the COM of the compound and that of the PLGA matrix restrained by using a biasing potential with a spring constant of 1000 kJ mol^–1^ nm^–2^. Calculations were performed using the SD integrator and a timestep of 2 fs. The weighted histogram analysis method (WHAM)^14^ was employed to combine the probability densities associated with sampled windows spanning the reaction coordinate and derive the free energy profile.

#### 2.1.4 Translocation Rate

The release *k_t_* of the hydrophobic DTXL and its conjugates from a hydrophobic polymer matrix into an aqueous solution can be described via a diffusivity–solubility model, which is commonly used to predict the permeation of hydrophobic drugs across membranes.^15a,15b^ Similarly, *k_t_* would mostly depend on the free energy of translocation variation, Δ*G*_t_^*^, between the preferred location of the drug (inside the matrix or at the matrix–W interface) and the reference value in the aqueous solution and, less extensively, on the diffusion coefficient *D* of the drug according to:^16^
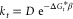
1where β is equal to 1/*k*_B_*T*, with *k*_B_ being Boltzmann’s constant and *T* the absolute temperature of the system.

The release kinetics of drugs from polymeric matrices has been extensively described by the general Ritger–Peppas equation:^17^
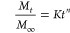
2where *M_t_* and *M*_∞_ are the cumulative amount of drug released at time *t* and at the infinite time, respectively, *n* is the diffusional exponent characteristic of the release mechanism, and *K* is a constant accounting for both the geometry of the problem and the drug–particle interaction strength.

#### 2.1.5 Aggregation Propensity

The propensity of the drug molecules to aggregate was measured at two different concentrations, namely, 9 and 27 mol. The system was set up using the GMX routine *gmx insert-molecules*. Initially, 1500 PLGA chains with length *N* = 10, 12,000 standard W molecules, 1700 antifreeze particles, and 27 PEG_25_–DTXL molecules were randomly mixed and dispersed inside a box having size 15 × 15 × 15 nm. The 27 PEG_25_–DTXL molecules were placed far apart by an average distance of *d* ∼5 nm between their respective DTXL moieties. For setting up the systems with PEG_10_–DTXL, OA–DTXL, and free DTXL, the drug beads in excess with respect to the PEG_25_–DTXL molecules were simply replaced by W to ensure the same exact starting configurations for all the tested cases. PBC were applied along the three directions. After a few nanoseconds, the PLGA and W phases separate and a biphasic system forms where freely diffusing drug molecules can redistribute in the two phases or at the interface depending on the relative solubility (see Figure S6). The cluster size was computed using the GMX routine *gmx clustersize* by measuring the number of contacts between drug beads within 0.6 nm over the 1 μs trajectory.

#### 2.1.6 Simulation Details

All simulations were performed using the GROningen MAchine for the Chemical Simulation (GROMACS) program (version 5.1.4).^18^ The modeled systems were first energy minimized using the steepest descent algorithm and then equilibrated in an NPT ensemble at constant number of molecules *N*, temperature *T*, and pressure *P*, using a Berendsen weak-coupling thermostat and barostat algorithms.^19^ In the production run, a constant temperature of 310 K, if not otherwise specified, was maintained using velocity-rescale thermostat^20^ with a time constant of 0.1 ps. The Parrinello–Rahman barostat^21^ was applied for isotropic pressure coupling with a reference pressure of 1.0 bar. The PME (particle mesh Ewald) method was used for computing long-range electrostatic interactions with a cutoff distance set to 1.4 nm, Fourier spacing of 0.12 nm, and cubic interpolation. The van der Waals interactions were cut off at 1.4 nm. The Verlet algorithm was employed to list all pairs within the cutoff, beyond which interactions could be ignored. The equations of motion were integrated using the leapfrog scheme except in the case of alchemical calculations where the SD integrator was used instead. Periodic boundary conditions were applied in the three dimensions. A timestep of 2 fs was used.

CG simulations were run using the MARTINI forcefield v2.2. Non-bonded interactions were shifted to zero with the use of potential modifiers at the cutoff distance of 1.1 nm. The combined use of the Verlet neighbor list algorithm and GROMACS v5 running on GPUs led to a good performance. Pressure and temperature coupling schemes were maintained similar to the atomistic simulations. The relative permittivity was set to 15, and a time step of 10 fs was used if not otherwise specified.

### 2.2 Materials and Experimental Methods

#### 2.2.1 Chemicals

Polydimethylsiloxane (PDMS) (SYLGARD 184) was purchased from Dow Corning Corp. (Midland, USA). Poly(vinyl alcohol) (PVA, Mw 31,000–50,000), poly(dl-lactide-co-glycolic) acid (PLGA, lactide:glycolide 50:50, Mw 38,000–54,000), poly(ethylene glycol) diacrylate (Mn 750) (PEG-DA), and 2-hydroxy-40-(2-hydroxyethoxy)-2-methylpropiophenone (photo-initiator) were purchased from Sigma-Aldrich (Missouri, USA). DTXL was purchased from Alfa Aesar (Massachusetts, USA). 4-(Dimethyl-amino)pyridine (DMAP) (99%) was purchased from Sigma-Aldrich. Dichloromethane anhydrous ≥99.8%, which contains 40–150 ppm amylene as stabilizer, was purchased from Sigma-Aldrich. Amine PEG 550 Da and 1000 Da were purchased from Creative PEGWorks.

#### 2.2.2 Synthesis of Discoidal Polymeric Nanoconstructs

Discoidal polymeric nanoconstructs (DPNs) were synthetized using a top-down fabrication strategy described previously by the authors.^22a,22b^ Briefly, the process involved the use of a PVA template, as a hydrophilic mold, presenting an ordinate pattern of cylindrical 1000 × 400 nm wells, which were carefully filled with a polymeric paste. Specifically, DPNs were synthetized using a mixture of PLGA and PEG-DA chains. Fifty milligrams of PLGA was dissolved in 1 mL of dichloromethane (CH_2_Cl_2_) and mixed with 6 mg of PEG-DA and any other molecule of interest (drug and prodrugs). Then, 0.6 mg of a photo-initiator (2-hydroxy-4′-(2-hydroxyethoxy)-2-methylpropiophenone) was added into the polymeric solution to allow the crosslinking of the PEGDA chains after exposure to UV light (366 nm). A fixed volume of polymeric mixture including OA–DTXL or PEG–DTXL (5 μL) was then spread through a blade over the PVA template to accurately fill each well. Finally, DPNs were purified via filtration and centrifugation.

#### 2.2.3 Synthesis of Succinic-DTXL

Derivatization of DTXL with ammine-PEG chains of different molecular weights required the prior formation of a DTXL intermediate product exposing a carboxylic group that was then reacted to succinic to realize succinic-DTXL (s-DTXL). Specifically, DTXL (50 mg, 6.19 × 10^–5^ mol) and 4-dimethylaminopyridine (DMAP) (12.38 × 10^–5^ mol) were dried under vacuum for 2 h before starting the reaction to completely remove any trace of humidity. Then, dried compounds were dissolved in 2 mL of anhydrous pyridine in a 25 mL round-bottom flask. The reagents were stirred for a few minutes in a nitrogen atmosphere at room temperature. Then, succinic anhydride dissolved in pyridine (1 eq) was added dropwise and left to react for 4 h under a nitrogen atmosphere at room temperature to produce the monosubstituted s-DTXL. The reaction mixture was washed three times with toluene and dried under low pressure to remove the pyridine and obtain a crystalline powder.

#### 2.2.4 Synthesis of PEG-DTXL

Pure succinic-DTXL (50 mg) was dissolved in anhydrous CH_2_Cl_2_ in a 50 mL round-bottom flask and activated using *N*′-ethylcarbodiimide hydrochloride (EDC) (1.2 eq) and *N*-hydroxysulfosuccinimide (NHS) (1.2 eq) and triethylamine (TEA) for 2 h under a nitrogen atmosphere at room temperature. Then, amine-PEG (550 or 1000 Da, 1 eq) was added into the reaction mixture and left overnight. The so-obtained raw product was washed with DCM and dried at low pressure to remove residual TEA. Then, it was dissolved in a minimal amount of CH_2_Cl_2_ and purified with automatic silica chromatography using a gradient of DCM (solvent A) and DCM:methanol (solvent B, 9:1).

#### 2.2.5 Synthesis of Oleic Acid–DTXL Prodrug

DTXL (50 mg, 6.19 × 10^–5^ mol) and DMAP (12.38 × 10^–5^ mol) were dissolved in 15 mL of anhydrous CH_2_Cl_2_ in a 50 mL round-bottom flask. The reagents were stirred for a few minutes in a nitrogen atmosphere at 0 °C. Then, oleoyl chloride (14.7 μL) was added dropwise to the mixture followed by stirring for 4 h under a nitrogen atmosphere and the temperature was maintained at 0 °C to produce the mono-substituted 2′-oleic-docetaxel. The conjugation of oleic-docetaxel was monitored by thin layer chromatography (TLC) using CH_2_Cl_2_:methanol (97:3) as a solvent. The formation of the DTXL conjugate was verified using ^1^H nuclear magnetic resonance (NMR). The reaction mixture was diluted with diethyl ether and washed first with 5% HCl and then using brine. The solution of 2′-oleoil-docetaxel was collected and filtered to remove the impurities. The product was finally dried under low pressure and stored at 0 °C. The collected solution was dissolved in CH_2_Cl_2_ and purified using a silica gel column with CH_2_Cl_2_:methanol (9:1) as the mobile phase to obtain the pure conjugate product.

#### 2.2.6 DPN Loading and Release Studies

The “direct loading” method was used to uniformly distribute and load the drug within the DPN polymeric matrix. Specifically, a 5 μL homogenous drug/polymer solution was uniformly spread using a blade over the surface of a 3 × 3 cm PVA template, containing about 10^8^ wells. The amount of drug loaded within DPN was calculated using an HPLC and reading the characteristic DTXL UV absorbance at 230 nm (Agilent 1260 Infinity, Germany). Samples for HPLC analysis were prepared by spinning down DPN at 12,700 RPM for 20 min, drying the pellet overnight, and dissolving the particles upon incubation with acetonitrile (ACN). Release studies were performed in a volume of 4 L of buffer at controlled pH 7.4 and 37 °C to reproduce the infinite sink conditions. At each time point, 200 μL of DPN solution was poured into Slide-A-Lyzer MINI Dialysis cups with a molecular cutoff of 10 kDa (Thermo Scientific) and dialyzed. At each time point, DPNs were collected and dissolved in ACN to read the amount of DTXL entrapped in the matrix.

## 3 Results

### 3.1 CG Model for PLGA

**Figure 1:**
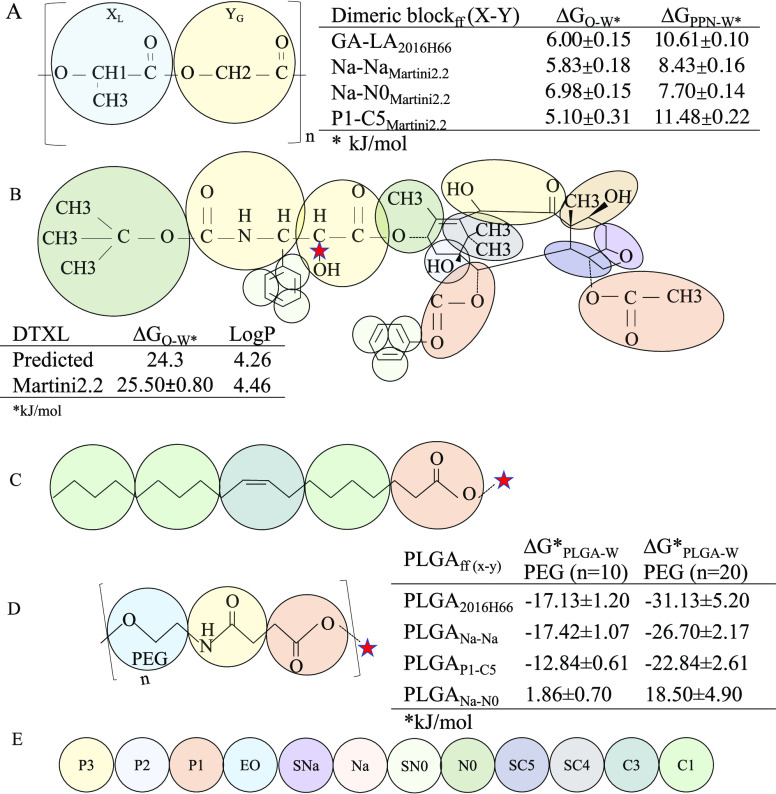
PLGA, DTXL, and DTXL-conjugate mapping at a coarse-grained resolution. (A) The repeating dimeric CG unit overlaps the chemical structure of a methyl-terminated PLGA chain (chain terminals are excluded from the UA to CG mapping). X and Y refer generically to any of the possible combinations of the considered MARTINI beads, namely, Na–Na, P1–C5, and Na–N0; the subscripts _L_ and _G_ refer, respectively, to the units with or without the chiral carbon, while the subscripts _DL_ and _LL_ refer to the two possible stereo-configurations of the chiral center. In the table (right) are shown free energies of transfer of a dimeric block (top left) from PPN (Δ*G*_PPN–W_) or O (Δ*G*_O–W_) to W solvent computed by thermodynamic integration (TI) at atomistic (UA-2016H66 forcefield) and coarse-grained (CG-MARTINI2.2 forcefield) resolutions with the three combinations Na–Na, P1–C5, and Na–N0. (B) CG beads overlap the chemical structure of the DTXL molecule. In the table are shown free energies of transfer of DTXL from O (Δ*G*_O–W_) to W computed by TI at the CG resolution, derived log *P*_ow_, and for a comparison, the experimental log *P*_ow_ value as reported by the European Medicines Agency. CG beads overlap the chemical structure of the oleic moiety (C) and PEG moiety (D), as used in this study. In the table is shown the polymer–W partition coefficient of the PEG chains computed by TI at the atomistic (2016H66) and coarse-grained (MARTINI) resolution with three alternative mapping schemes (Na–Na, P1–C5, and Na–N0) for two different degrees of polymerization (*N* = 10, 20). In (E) are shown scale colors of CG beads based on the associated MARTINI type.

Following a previous work by the authors on the atomistic modeling of PLGA and PEG,^5^ first a CG model for PLGA was defined. This polymer, which is extensively used within the drug delivery and nanomedicine community, is represented as a sequence of dimeric units of lactic and glycolic acid monomers (LA/GA 50:50), with LA in alternated l and d configurations. Invoking standard MARTINI parametrization v2.2, three possible CG dimeric blocks were considered, namely, Na–Na, Na–N0, and P1–C5. To identify the best dimer block, the PLGA solubility in O, W, and PPN was assessed for all three configurations above and compared with the results at an atomistic resolution. Specifically, the free energy of transfer Δ*G* from PPN to W (Δ*G*_PPN-W_) and from O to W (Δ*G*_O–W_) for the three CG models were compared with the same energy values calculated with the UA model GA-LA_2016H66_. Data are tabulated in Figure 1A showing that the Na–Na dimer block returned values that are closer to the atomistic model as compared to the other two CG models.

The determination of the bonded terms (bond, angles, and dihedrals) between adjacent CG beads was achieved after matching via an iterative procedure the bond distributions extracted from CG and UA simulations from a previous work.^5^ For the PLGA parametrization, 40 chains with *N* = 16 were simulated for 300 ns at 330 K, which is right above the PLGA experimental glass-like transition temperature (315–320 K),^23^ to favor chain mobility and improve the sampling of the bond distributions. Considering the long relaxation time of the PLGA chains in a homopolymer melt, the bond distributions of a PLGA chain with *N* = 64 was also sampled in PPN for 100 ns at 300 K. The atomistic trajectories were converted into a CG trajectory by assigning the COM of the designated group of atoms to MARTINI beads according to the mapping strategy defined above. Bond and angle distributions between COMs of selected bonded beads were extracted from the target atomistic trajectory using the GROMACS routines “gmx distance” and “gmx angle”, respectively. The computed average distances and angles were thus chosen as equilibrium lengths and angles for harmonic and harmonic cosine functions describing the bond, angle, and dihedral interactions between CG beads, respectively (the equations describing bonded interactions are reported in Figure S1A). The force constants in the same equations were chosen to reproduce the widths of the target atomistic distributions. CG parameters were adjusted by trial and error to reduce the mismatch between CG and UA distributions (Figure S1A,B). The stereo-regular effects associated with the presence of both d and l enantiomers of lactic acid were integrated into the calibration of dihedral angles.

Specific properties of the polymer, such as the density δ, gyration radius *R*_g_, and end-to-end distance *R*_e_, were considered to assess the accuracy of the CG model for PLGA. Results obtained at the CG level using the three mapping schemes above ((S)Na–Na, Na–N0, P1–C5) were systematically compared with data obtained using the atomistic model. For the release studies below, we chose to represent PLGA using the Na–Na mapping scheme, which reasonably reproduced properties of interest for this work (see SI3 for the discussion on the model accuracy).

### 3.2 CG Models for DTXL and Its Conjugates

The chemotherapeutic molecule DTXL was considered as a test drug,^9^ both in its free form and conjugated to other molecules, like OA and PEG chains with two different degrees of polymerization (*N* = 10 and 25). The topologies for PEG and oleic moieties were readily available from the MARTINI website, whereas the models for the DTXL compound and its linkers were built following the MARTINI approach. As per the PLGA model, the choice of CG beads was based on the reproducibility at the CG resolution of the experimental partition coefficient in O/W log *P*_ow_ (Figure 1). In the case of linkers, bead types were assigned to reflect the thermodynamic behavior of the chemical groups parametrized according to the MARTINI forcefield v2.2.

In the case of DTXL (Figure 1B), beads of standard size were used to map four to five heavy atoms, whereas small-size beads were chosen to map groups of atoms in ring structures. Bonded terms were automatically assigned via the use of PyCGTOOL^24^ based on the consensus between bond, angle, and dihedral distributions derived from CG and atomistic simulations of the molecule dissolved in O at 300 K (Figure S8). The resulting log *P*_ow_ for DTXL was estimated to be 4.4, which is close to the experimental value of 4.2.

### 3.3 Release of DTXL and Its Conjugates from a PLGA Matrix—CG Model

The US method was employed to compute the energetic profile of compounds translocating across a PLGA–W system. The free energy minima identify regions where compounds preferentially partition: the deeper the free-energy well is, the more stable the partition is. The translocation free energy Δ*G*_t_^*^ indicates the amount of energy required to move the drug from the preferred partition region into the aqueous solution surrounding the PLGA matrix. The higher the Δ*G*_t_^*^, the slower the drug release kinetics.

**Figure 2:**
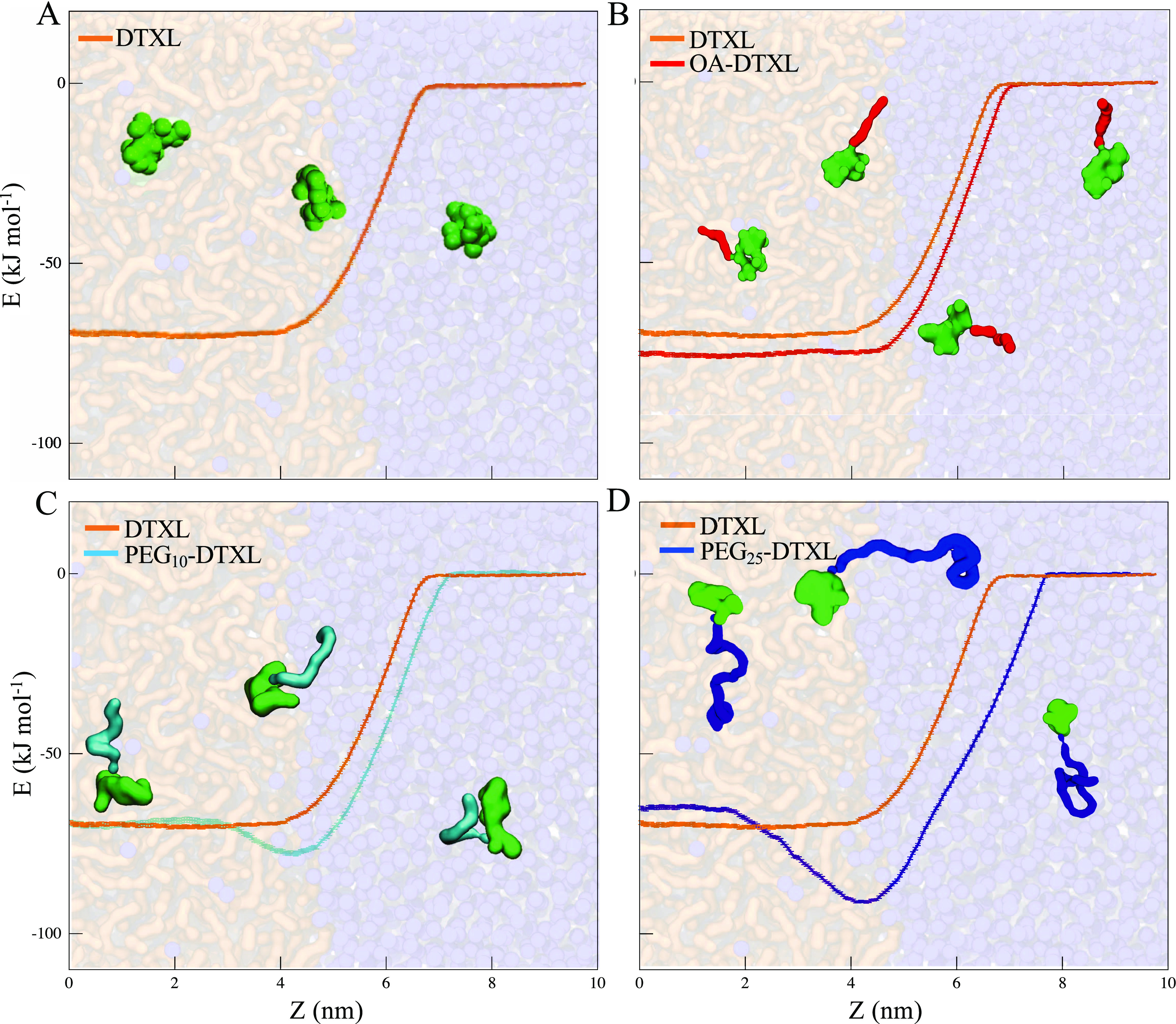
Translocation of DTXL and DTXL conjugates from a polymeric matrix to an aqueous solution. Predicted free energy of translocation of (A). DTXL (orange), (B) OA–DTXL (red), (C) PEG_10_–DTXL (cyan), and (D) PEG_25_–DTXL (blue) molecules, from a poorly hydrated PLGA matrix (transparent orange, left), made of *N* = 10 chains, to an aqueous solution (transparent blue, right).

Figure 2A shows the energetic profile associated with the hydrophobic molecule DTXL. A free energy minimum within the matrix suggests its preferential partition within PLGA as opposed to W, with a free energy barrier Δ*G*_t_^*^ of about −69.8 kJ mol^–1^. When a short PEG moiety with *N* = 10 is attached to the DTXL molecule, the corresponding conjugate partitions more stably at the PLGA–W interface as demonstrated by the minimum in energy profile (Figure 2C), with a corresponding translocation free energy barrier Δ*G*_t_^*^ of −76.9 kJ mol^–1^. If a longer PEG chain with *N* = 25 is conjugated to the DTXL molecule, the resulting molecule shows a deeper minimum at the PLGA–W interface, with a corresponding translocation barrier Δ*G*_t_^*^ of −90.7 kJ mol^–1^ (Figure 2D). These results would suggest a stronger propensity of the PEG–DTXL prodrugs to accumulate at the PLGA–W interface, with PEG_25_–DTXL being more stable than PEG_10_–DTXL. This appears to be in line with previous reported observations for which homopolymers can act as surface active substances.^25^ Finally, OA–DTXL presents a slightly higher preference to stay in the matrix than free DTXL with a translocation free energy barrier Δ*G*_t_^*^ of −75.3 kJ mol^–1^ (Figure 2B).

**Figure 3:**
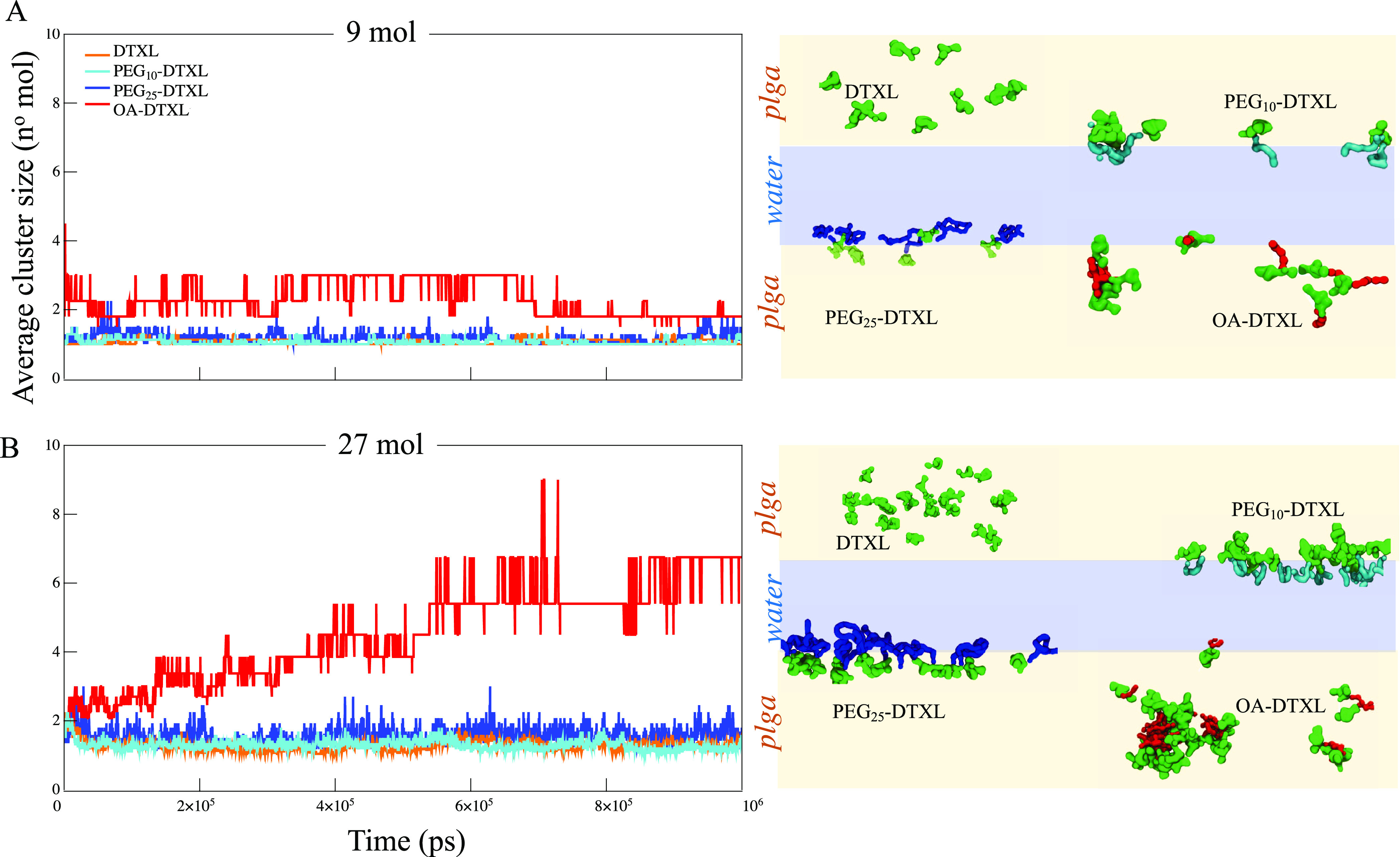
Aggregation propensity of the DTXL conjugates. In the left column, plots present the average size of clusters of DTXL (orange profile), OA–DTXL (red profile), PEG_10_–DTXL (cyan profile), and PEG_25_–DTXL (blue profile) molecules formed over time within a PLGA–W mixture, for two different DTXL concentrations: (A) 9 and (B) 27 molecules, respectively. In the right column, snapshots of the molecular clusters at equilibrium are represented, where light blue indicates the aqueous solution and light orange refers to the PLGA matrix.

Then, the aggregation propensity of the four molecules with the PLGA matrix was predicted at two different concentrations, namely, 9 and 27 mol. As shown in Figure 3, OA–DTXL had a strong propensity to aggregate, in a concentration-dependent manner. This behavior was not observed for the other three compounds DTXL, PEG_10_–DTXL, and PEG_25_–DTXL, all of which were stable as monomers in the two tested configurations (Figure 3). This result is not surprising considering that, among all the four tested compounds, OA–DTXL is the most hydrophobic molecule (log P≈9) and is even more hydrophobic than PLGA (log P ≈4). Note also that the present computational setup and the free energy calculations have all been performed assuming a drug concentration well below the critical conditions for aggregation. As such, data of Figure 2 are not affected by any possible aggregation.

### 3.4 Release of DTXL and Its Conjugates from a PLGA Matrix—Experimental Release Profiles

**Figure 4:**
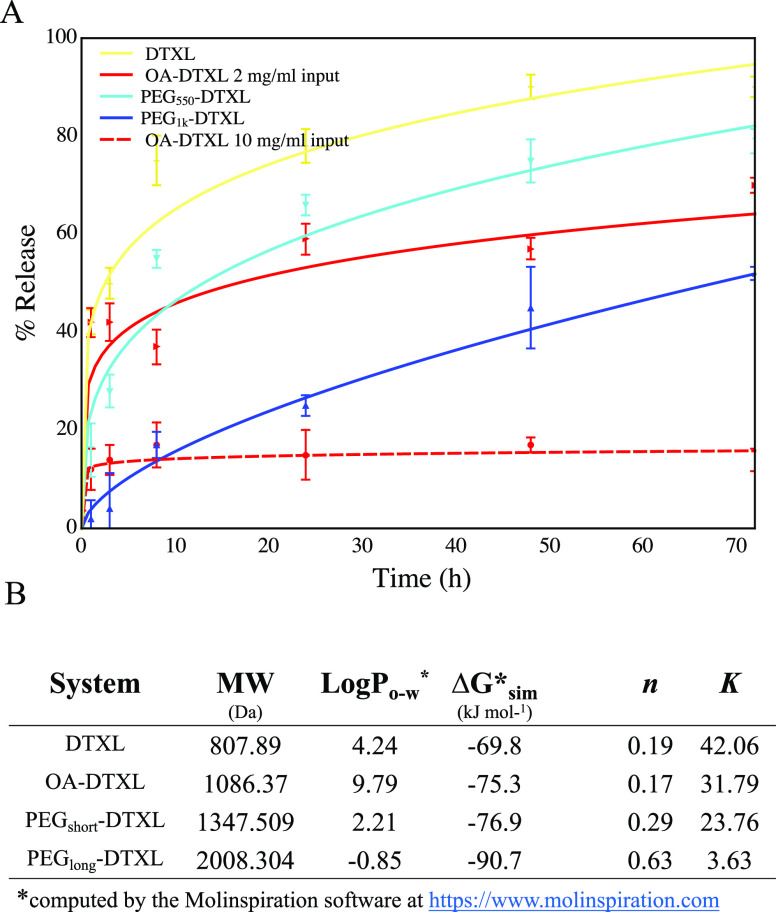
Release kinetics of the DTXL conjugates. (A) Experimental release profiles of DTXL and its conjugates from a PLGA matrix to an aqueous solution over a period of 72 h (DTXL, yellow line; OA–DTXL at 2 mg, red solid line; OA–DTXL at 10 mg, red dashed line; PEG_10_–DTXL, cyan line; PEG_25_–DTXL, blue line). (B) Molecular weight (MW), partition coefficient in O–W solvents log *P*_ow_ computed by Molinspiration software, and free energy barriers ΔG* across the matrix/solution interface, as determined from the simulations (sim) (see Methods). We report the values for *K* and the diffusional exponent characteristic *n* derived by fitting eq 2 with the experimental data.

The experimental release profiles of DTXL, PEG_10_–DTXL, PEG_25_–DTXL, and OA–DTXL from a PLGA particle to an aqueous solution are presented in Figure 4. For DTXL and its conjugates, amounts of drug released are examined in the first 72 h (Figure 4A). Note that within this time frame, the release is mostly diffusion driven as the PLGA matrix is still intact. DTXL has the fastest experimental release kinetics, corresponding to 75 ± 5% of drug released within the first 8 h and reaching almost 90% release at 72 h of incubation (Figure 4A, yellow line). Not surprisingly therefore, DTXL is associated with the smallest predicted polymer–solvent translocation free energy barrier (Figure 4B), returning a Δ*G*_t_^*^ = −69.8 kJ mol^–1^. The experimental release kinetics of PEG_550_–DTXL is slower than that of DTXL (Figure 4A, cyan line), reaching 55 ± 1.8% of drug released in the first 8 h. This agrees with a higher energy required for translocation, which is predicted to be Δ*G*_t_^*^ = −76.9 kJ mol^–1^ (Figure 4B). Moving from a short to a long PEG configuration, the mobility of the conjugates decreases as well as its release rate. Indeed, the experimental release kinetics of PEG_1k_–DTXL (MW 1000 Da) is even slower than that of PEG_550_–DTXL. It takes approximately 72 h for releasing 52 ± 1.4% of the loaded drug (Figure 4A, blue line). Consistently, the predicted translocation free energy barrier Δ*G*_t_^*^ is higher, returning a value of Δ*G*_t_^*^ = −90.7 kJ mol^–1^ (Figure 4B).

Finally, the release for the OA–DTXL conjugates rapidly increases in the first few hours reaching a value of ∼40% at 8 h (Figure 4A, solid red line), which is comparable with that of PEG_550_–DTXL cases (55 ± 1.8%). Following this phase, the release rate for OA–DTXL reduces to return a ∼70% release at 72 h. Throughout the observation period, OA–DTXL presents a release kinetics that is comparable with that of the short PEG, and this is indeed confirmed also by the CG prediction that estimates a Δ*G*_t_^*^ = −75.3 kJ mol^–1^ (Figure 4B). Note that the difference between Δ*G*_t_^*^ for OA–DTXL and PEG_550_–DTXL is only by ∼2%. The slightly slower release observed experimentally for OA–DTXL as opposed to PEG_550_–DTXL could be associated with a partial aggregation of the more hydrophobic OA–DTXL molecules. It is indeed reasonable to hypothesize that upon initial diffusion from the PLGA matrix toward the aqueous solvent, OA–DTXL could accumulate and aggregate at the polymer–W interface. Notably, the presence of OA–DTXL clusters at the PLGA particle–W interface was evidenced by TEM images (Figure S7). Another possibility is that the release kinetics of OA–DTXL is dominated by the slower diffusion within the matrix of OA–DTXL aggregates formed during the loading procedure. Clusters of OA–DTXL would diffuse much less than their molecular counterparts, as shown in Figure 4A by comparing the experimental release profile for low-concentration OA–DTXL (solid red line) and high-concentration OA–DTXL (dashed red line). Indeed, the actual behavior could result from the combination of the two different conditions described above. In perspective, thermodynamic quantities estimated via MD simulations can feed analytical expressions for predicting the release kinetics.

### 3.5 Limitations and Future Directions

The proposed CG framework is general and could be applied to any drug with a stronger affinity for the selected delivery platform as compared to the local environment. In this work, we have selected the PLGA matrix and DTXL derivatives as a case study. Indeed, both PLGA and DTXL are extensively used in the field of drug delivery and nanomedicine. The results reported are independent of different geometrical attributes and polymer concentrations and molecular weights. Here, the physicochemical features of the PLGA matrix (molecular weights and concentrations) are kept constant, as the observation time (72 h) is sufficiently short to assume no significant polymer degradation. In the case of a sustained drug release, over a prolonged period of time (weeks and months), the drug translocation barrier must also reflect morphological changes of the matrix and variations in drug solubility (e.g., as polymer biodegradation progresses, the polymer chains shorten and the matrix hydration increases). The approach can be extended to the study of the translocation of drugs encapsulated in heterogenous matrices of mixed composition and higher structural complexity.

A more faithful representation of the release mechanism should also include the contribution of the drug self-diffusion coefficient within the polymer matrix. However, this is difficult to accurately describe at this level of representation. In fact, coarse graining makes the energy landscape smoother, speeding up the kinetics of the system. With MARTINI 2, the speed-up factor in the diffusional dynamics of different compounds can be difficult to predict.^26^ Considering that the diffusive dynamics depends on the mass of the compounds, MARTINI 3 is expected to be more accurate as it uses smaller beads.^27^ In MARTINI 3, partial charges can also be included to obtain more reliable results, as these are known to affect interactions between atoms and solvation free energies. Indeed, higher accuracies could be obtained at an all-atom resolution, at the cost of computational time. Moreover, drug–drug interactions have been neglected and these could affect the diffusive dynamics of compounds when aggregates are formed. Yet, the proposed approach shows the potential for assessing the release of molecules from polymeric matrices. Simulations can help predict the impact of cluster size and shape in the release mechanism of the drug out of the matrix. After having established the relative difference in drug–drug and drug–polymer affinities, the translocation barrier of the drug in its aggregated form can be predicted.

## 4 Conclusions

A CG MARTINI model for PLGA, DTXL, and DTXL conjugates was built and validated against all-atom models. In the case of PLGA, structural and solubility properties relevant for mass transport problems were determined as well as the relative miscibility with PEG chains at varying concentration ratios and molecular weights. Then, the retention of DTXL and its conjugates with varying solubilities (from the most hydrophobic OA to the hydrophilic PEG polymer with two different lengths) within the PLGA matrix was predicted. Finally, correlations between the experimental release rates and translocation energies Δ*G*_t_^*^ from the PLGA matrix were assessed. Fast release kinetics properly corresponded to lower translocation barriers Δ*G*_t_^*^. In addition, this study provides evidence of molecular partitioning, especially in the case of PEG–DTXL conjugates, at the matrix–W interface. The same approach is therefore useful for predicting the orientation and stability of ligands on the particle shell for optimization of the particle coating.

Although further improvements in terms of model accuracy could be implemented, this work demonstrates that thermodynamic quantities estimated via MD simulations can help anticipate drug release kinetics from PLGA matrices, minimizing experimental workload and cost.

Most of the analytical and computations models used to predict the release profile of drugs need experimental data as inputs (e.g., drug diffusivity). However, we should aim at reducing the experimental workload and cost. Thermodynamics and kinetics quantities estimated via simulations can be a valuable alternative to feed analytical equations instead.

The proposed approach could be used to predict the pharmacological behavior of PLGA implants loaded with a variety of payloads under different conditions.

## Supporting Information Available

Supporting methods: PLGA polymer chain and PLGA/PEG miscibility (setup and properties); supporting results: discussion about the validation and accuracy of the coarse-grained model for PLGA (density, gyration radius, end-to-end distance, and PEG-PLGA miscibility); coarse-grained model for PLGA: model validation, setup for umbrella sampling simulations, setup for aggregation propensity, experimental setup, and TEM images of DPN loaded with docetaxel conjugates and coarse-grained mapping of docetaxel (PDF)
