# Supplementary material for: A Coarse-Grained Molecular Dynamics Description of Docetaxel-Conjugate Release from PLGA Matrices
Source: Biomacromolecules. 2022 Oct 14;23(11):4678–86. doi: 10.1021/acs.biomac.2c00903 (PMC9667470; doi:10.1021/acs.biomac.2c00903)
Supplement: Supplementary file 1 — bm2c00903_si_001.pdf [file bm2c00903_si_001.pdf]

SUPPORTING INFORMATION

A COARSE-GRAINED MOLECULAR DYNAMICS DESCRIPTION OF DOCETAXEL-CONJUGATE RELEASE FROM PLGA MATRICES

Martina Pannuzzo[*♣], Alessia Felici [♣], and Paolo Decuzzi[*]

Laboratory of Nanotechnology for Precision Medicine

Fondazione Istituto Italiano di Tecnologia,

Via Morego 30, Genoa 16163, Italy

* Corresponding author: martina.pannuzzo@gmail.com, paolo.decuzzi@iit.it

♣ Current affiliations:

Martina Pannuzzo, PhD – R&D Center, PharmCADD, Busan Republic of Korea, 48060

Alessia Felici, PhD – Department of Medicine, Stanford University, Stanford (CA – USA), 94305

Supporting Methods and Results

S1.1. Polymer chain properties and setup. The radius of gyration R_g of the polymer chain is defined as the root mean square of the distance of all n atoms from their COM (center of mass) according to

$$R_g{}^2 = \frac{1}{2}\sum_{i=1}^{n}(r_i - r_{COM})^2 \quad (1)$$

where r_i and r_{COM} denote the position vectors of the first and COM atoms in the polymer chain, respectively. The end-to-end distance R_e, between the start and the end of the polymer, is defined according to

$$\langle R_e{}^2\rangle^{1/2} = \langle(r_n - r_1)^2\rangle^{1/2} \quad (2)$$

where r_n denotes the position vector of the last atom in the polymer chain. The angular brackets ⟨...⟩ indicate the mean value over the polymer molecules and simulations timeframes. According to the Flory–Huggins theory, the gyration radius R_g of a polymer chain in a dilute solution scales as $R_g \propto N^v$, where N is the number of monomers per chain (degree of polymerization) while v reflects the dependence of the polymer size on the solvent quality. In a good solvent (v = 3/5), the polymer swells due to the energetically more favorable monomer-solvent interaction. In a bad solvent (v = 1/3), the polymer collapses as the monomer-monomer attraction prevails over monomer-solvent interactions. Finally, in a theta Θ solvent (v = 1/2), the polymer behaves as an ideal chain (random walk coil) due to comparable monomer-monomer and monomer-solvent interactions.[1]

S1.2 PLGA/PEG miscibility. The PLGA/PEG miscibility trend was measured by looking at the number of interspecies contacts c in the mixtures. This quantity was estimated over time using the GROMACS routine "gmx mindist", setting a threshold distance of 0.6 nm. The number c was next normalized by the theoretical maximum number of contacts c_{max} that PEG could form with PLGA, considering that in a simple cubic lattice each bead of the polymer chain has 6 nearest neighbors (coordination number z = 6 according to Flory's theory), thus middle and terminal beads of a chain

can interact with up to 4 and 5 beads of a different chain, respectively. Normalized extreme values $c = c_{max}$ and $c = 0$ would respectively indicate full PLGA-PEG miscibility or segregation.

S2. Coarse-Grained model for PLGA: model validation

A

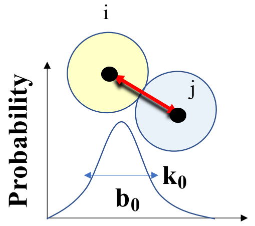

BOND		b_0 (nm)	k_b(kJ mol^{-1} nm^{-2})
X_L	Y_G	0.377	25000
Y_G	X_L	0.352	25000

ANGLES			θ_0 (deg)	k_0(kJ mol^{-1})
X_L	Y_G	X_L	133.5	30.0
Y_G	X_L	Y_G	109.0	50.0

DIHEDRALS				ϕ_s (deg)	k_ϕ (kJ mol^{-1})
X_{LD}	Y_G	X_{LL}	Y_G	-70.0	2.2^1
Y_G	X_{LL}	Y_G	X_{LD}	130	0.5^2
Y_G	X_{LL}	Y_G	X_{LD}	180	0.5^1, 0.2^3
X_{LL}	Y_G	X_{LD}	Y_G	70.0	2.2^1
Y_G	X_{LD}	Y_G	X_{LL}	-130	0.5^2
Y_G	X_{LD}	Y_G	X_{LL}	180	0.5^1, 0.2^3

The superscript refers to the nth multiplicity.

B

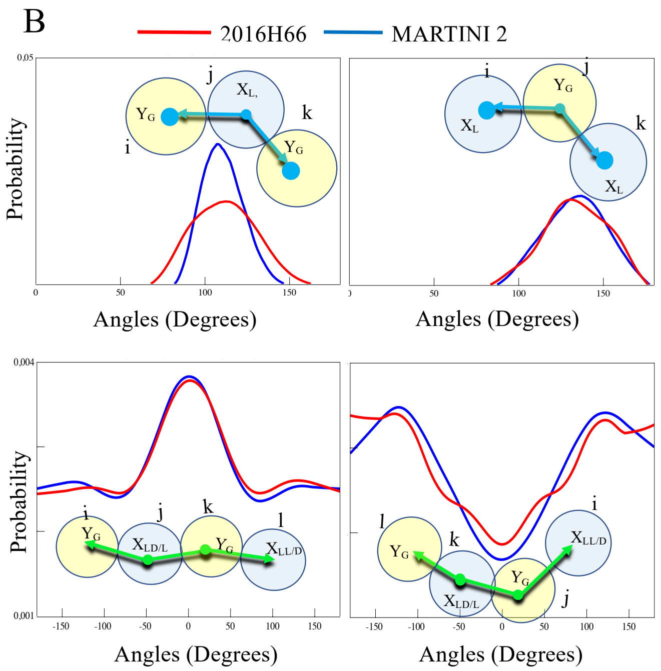

**Bond Type 1*: $V_b(\mathbf{r}_{ij}) = \frac{1}{2} \boldsymbol{k}_{ij}^{b}(r_{ij} - \mathbf{b}_{ij})^2$

**Angle Type 2*: $V_a(\boldsymbol{\theta}_{ijk}) = \frac{1}{2} \boldsymbol{k}_{ijk}^{\boldsymbol{\theta}}(\cos(\boldsymbol{\theta}_{ijk}) - \cos(\boldsymbol{\theta}_{ijk}^{0}))^2$

**Proper Dihedral Type 1*: $V_d(\boldsymbol{\phi}_{ijkl}) = \frac{1}{2} \boldsymbol{k}_{\phi}(1 + \cos(n\phi - \boldsymbol{\phi}_s))$

Figure S1. **PLGA Coarse-Grained Mapping. A**. Bond, angle, and dihedral parameters between CG beads defined according to the definition of the MARTINI forcefield. **B.** Angle and dihedral distributions at atomistic (red line) and coarse-grained resolution (blue line).

S3. Accuracy of the Coarse-Grained model for PLGA. Specific properties of the polymer, such as the density δ, gyration radius R_g, end-to-end distance R_e, were considered to assess the accuracy of the CG model for PLGA. Note that these physico-chemical properties would significantly affect the transport dynamics of small molecules within a PLGA matrix, thus their reproducibility by the CG model is key. The density δ and chain properties of pure polymer samples consisting of 40

PLGA chains were first quantified for two different degrees of polymerization, namely N = 16 and 64. Results obtained at the coarse-grained level using the three mapping schemes above ((S)Na-Na, Na-N0, P1-C5) were systematically compared with data obtained using the atomistic model (**Table S1**). The density values for the pure $PLGA_{16}$ samples were 1.35±0.003 and 1.217±0.05 g/cm^3 at CG and UA resolution, respectively. For higher molecular weights, $PLGA_{64}$, samples retuned slight larger values with δ equal to 1.37±0.07 and 1.27±0.003 g/cm^3 at CG and UA resolution, respectively. Overall the difference between the CG and UA predictions amounts to ~ 0.1 g/cm^3, which is <10%. Notably, such a small difference was also documented for the other two parameters, radius of gyration R_g and end-to-end distance R_e, in the case of $PLGA_{16}$ and $PLGA_{64}$ samples, regardless of the mapping type (**Table S1**). Multiple factors could contribute to explain this minor discrepancy. According to the 4:1 MARTINI mapping scheme (v2.2), 4 heavy atoms in the atomistic model are represented by one bead in the coarse-grained model. In our model, a standard size CG bead maps instead the 5 heavy atoms of the group X_L. Additionally, the standard size MARTINI bead has a larger radius than the chemical group X_G that it accommodates. This in turn causes a slight interpenetration of neighboring CG beads when their bond length is assigned to map the atomistic distance between X_G and X_L COMs, promoting an artificial increased beads affinity and a partial collapse of the chain. Finally, the methyl group in the LA monomer likely disfavors chain packing as the experimental increased density of PLGA with the glycolic content suggest[2], but this effect is expected to smooth when the system is modeled at CG resolution. As an alternative, a smaller size bead (S type) was employed to map the X_G monomer. When PLGA chains were built using a dimeric SNa-Na unit, the resulting density values for pure $PLGA_{16}$ and $PLGA_{64}$ coarse-grained samples were approximately equal to ≈1.1, which is 0.1 g cm^{-3} lower than the estimated values from atomistic simulations of similar systems configurations (see **Table S1**).

Homopolymer melt	Density at 330K (g/cm^3)	Chain Gyration Radius (nm)	Chain End-to-End Distance (nm)
**PLGA$_{UA_16}$**	1.21±0.05, 1.3*	0.87±0.02	2.17±0.07
**PLGA$_{Na\text{-}Na_16}$**	1.35 ±0.003 (~+10%)	0.87±0.03	2.19±0.10
**PLGA$_{SNa\text{-}Na_16}$**	1.15±0.005 (~-5%)	0.86±0.03	2.16±0.10
**PLGA$_{P1\text{-}C5_16}$**	1.34±0.003 (~+10%)	0.87±0.03	2.19±0.10
**PLGA$_{Na\text{-}N0_16}$**	1.33±0.001 (~+9%)	0.87±0.03	2.19±0.10
**PLGA$_{UA_64}$**	1.27±0.003	1.84±0.003	4.67±0.03
**PLGA$_{Na\text{-}Na_64}$**	1.37±0.07 (~+8%)	1.86±0.02	4.58±0.19
**PLGA$_{SNa\text{-}Na_64}$**	1.17±0.005 (~-8%)	1.91±0.02 (~+3%)	4.5±0.10
**PLGA$_{P1\text{-}C5_64}$**	1.36±0.004 (~+7%)	1.93±0.03 (~+5%)	4.85±0.20
**PLGA$_{Na\text{-}N0_64}$**	1.35±0.003 (~+6%)	1.87±0.03	4.71±0.14
* At 300K			

Table S1. PLGA physico-chemical properties. Density, Gyration Radius and End-to-End distance for a PLGA chain built from the dimeric (S)Na-Na, P1-C5, and Na-N0 units. The percentage difference between the results derived from the CG simulations and the atomistic simulations (UA) are listed in brackets when significant.

Next, the solubility of PLGA in selected solvents was assessed. According to the Flory theory, as detailed in the Methods section S1.1, the size of a polymer chain in a dilute solution depends on the solvent: the polymer swells if the polymer-solvent interactions are dominating (good solvent condition); while the polymer collapses if the polymer-polymer interactions are dominating (poor solvent condition). A PLGA chain with increasing degree of polymerization (N = 16, 37, 64 and 87) was simulated in three different solvents, namely acetone (PPN), octanol (OTL) and water (W), at 300 K for 300 ns and 1 μs at atomistic and CG resolution, respectively. The gyration radius R_g was estimated over the equilibrated sampled trajectory and analyzed as a function of the PLGA degree of polymerization N (**Figure S2**). The exponent υ in the relationship $R_g \approx N^{\upsilon}$ was estimated by a least-square fitting of the numerical data. The solubility trend observed at the atomistic level was qualitatively reproduced at a CG resolution: water and octanol are poor solvents (υ values below 1/3)

unlike acetone (υ values above 1/2) for PLGA. A υ value lower than 1/3 for a polymer dissolved in water or acetone has been attributed to finite size effects and already discussed elsewhere by the authors[3]. According to the empirical "like dissolves like" rule, an ideal solubility of PLGA in acetone would have been expected for the Na-Na dimer model, considering also that the chain and the solvent are both made by Na type beads. This unexpected, suboptimal solubility of PLGA in acetone at the CG level (υ =0.44 ± 0.01), conversely to the atomistic case (υ = 0.58 ± 0.003), must be attributed to the slight interpenetration of adjacent beads, as described above, that would cause a partial collapse of the chain. The discrepancies between the values estimated at atomistic and coarse-grained levels are narrow for PLGA chains modeled with a dimeric P1-C5 unit (**Figure S2C**), as compared to the alternative Na-Na and Na-N0 cases (**Figures S2B** and S**2D**). The more hydrophilic P1 beads, as compared to the Na type beads, likely contributed to reduce the artificial intrachain higher affinity and the partial collapse of the chain.

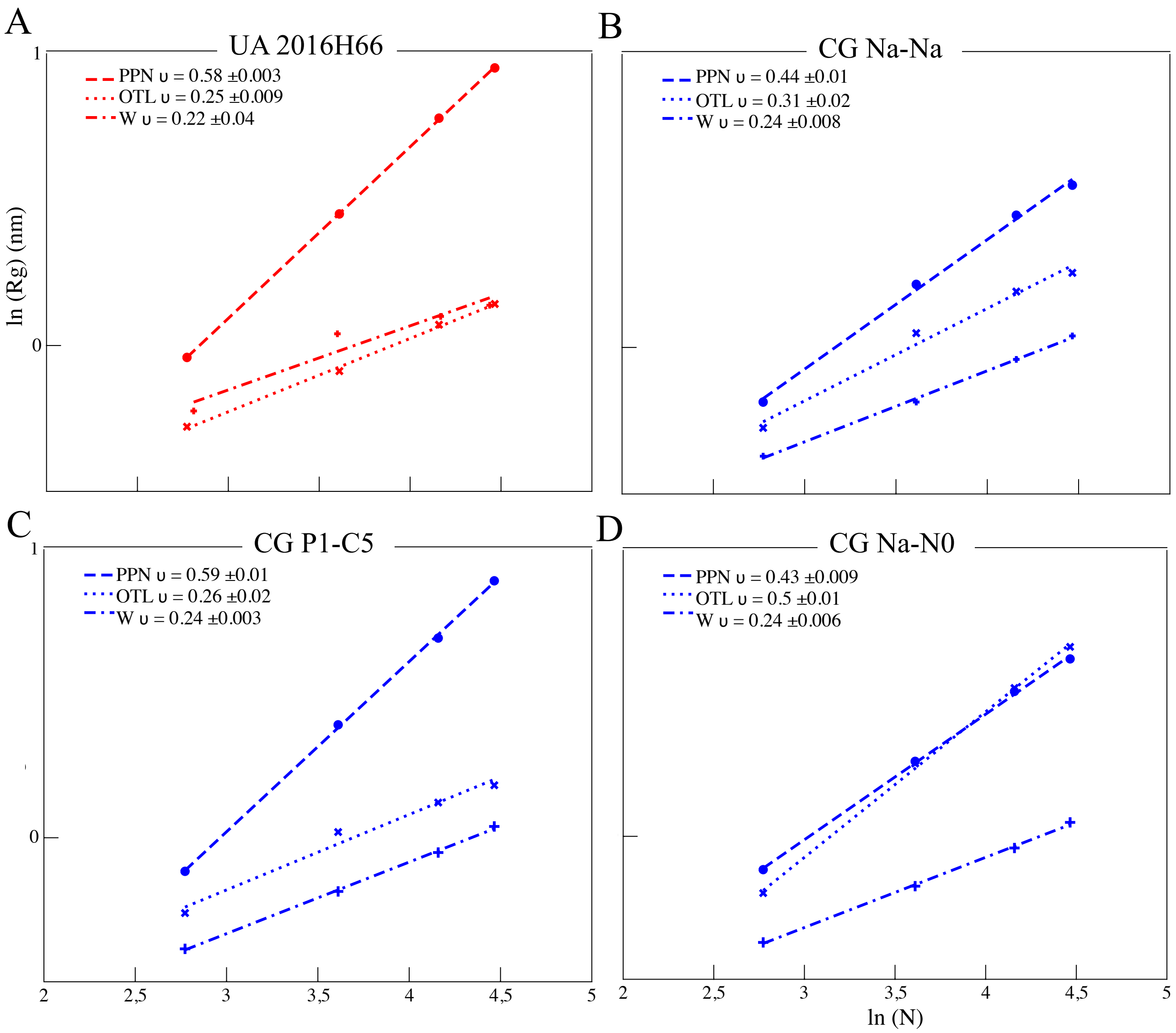

Figure S2. PLGA radius of gyration versus degree of polymerization at atomistic and coarse-grained resolutions. Double-logarithmic plot of Radius of gyration ln (R_g) as a function of the degree of polymerization ln(N) for PLGA in water (dash-dot line), acetone (dashed line), and octanol (dot line) solvents modeled at UA (**A**) and CG resolution with three mapping schemes, namely Na-Na (**B**), Na-N0 (**C**), and P1-C5 (**D**) units. The exponent υ in the relationship $R_g \approx N^{\upsilon}$ coincides with the slope of the least square-fitted lines.

Finally, the miscibility of PLGA and PEG was also estimated at CG level and compared with the atomistic results. First, the three alternative mapping schemes for PLGA were tested to check for the

reproducibility of the PLGA–water partition coefficient of PEG chains with degrees of polymerization $N = 10$ and 20 estimated at atomistic resolution (see table in **Figure 1D**). Next, four different mixtures were generated after combining PLGA chains having degree of polymerization $N = 64$ with PEG chains at two degrees of polymerization $N = 16$ and 64, and at two different ratios ϕ, namely 8:92 and 46:54, similarly as previously done at atomistic scale.[4]. The miscibility trend was measured by looking at the interspecies contacts in the four binary mixtures at atomistic and CG resolution in the four binary mixtures: A) $8PEG_{16}$:$92PLGA_{64}$ consisting of 92% of PLGA chains having degree of polymerization N=64 randomly mixed with 8% PEG chains with degree of polymerization N=16; B) $8PEG_{64}$:$92PLGA_{64}$ consisting of 92% of PLGA chains having degree of polymerization N=64 randomly mixed with 8% PEG chains with degree of polymerization N=64; C) $46PEG_{16}$:$54PLGA_{64}$ consisting of 54% of PLGA chains having degree of polymerization N=64 randomly mixed with 46% PEG chains with degree of polymerization N=16; D) $46PEG_{64}$:$64PLGA_{64}$ consisting of 54% of PLGA chains having degree of polymerization N=64 randomly mixed with 46% PEG chains with degree of polymerization N=64;

The normalized number of interspecies contacts along 1 μs for the 4 configurations, as previously computed from atomistic simulations[3], was accurately reproduced at the CG level by the Na-Na mapping scheme (**Figure S3**): at a lower PEG concentration, PLGA blends well with both shorter PEG_{16} and longer PEG_{64} chains (systems A and B), the miscibility partially reduces at higher concentration of shorter PEG_{16} chains (systems C), and segregation occurs in the system with the higher concentration of longer PEG_{64} chains (systems D). Visual evidence of the four PLGA/PEG miscibility regimes as a function of polymers ratio (Φ_{PEG}=8%, 46%) and length (N_{PEG}=16, 64) is reported in **Figure S3**.

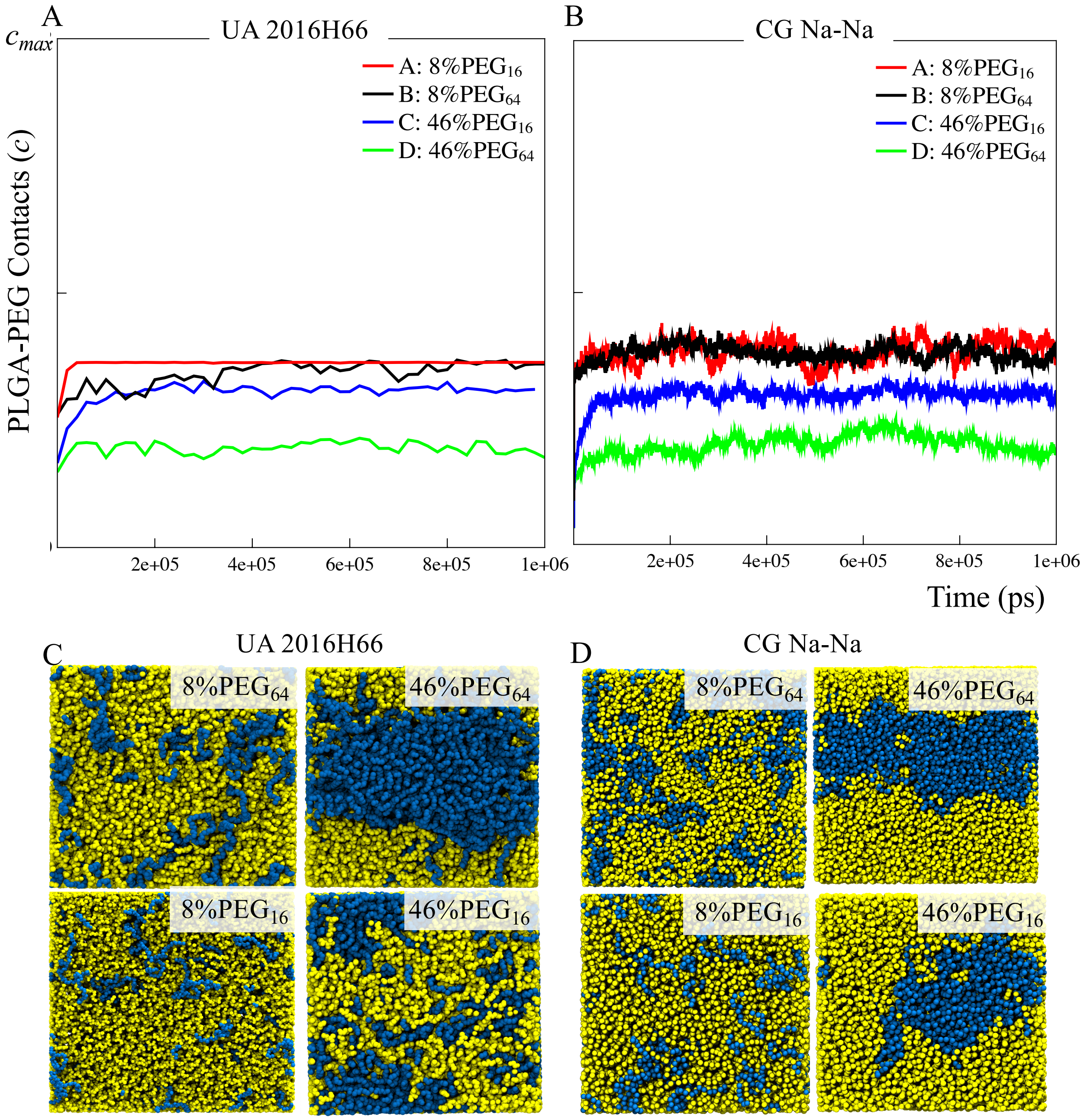

Figure S3. PLGA/PEG miscibility at atomistic and coarse-grained resolutions. Normalized number of PLGA/PEG contacts computed over 1 μs at atomistic (**A**) and CG resolution (Na-Na mapping scheme) (**B**), reported as a function of PEG:PLGA concentration ratio (Φ_{PEG} = 8%, 46%) and PEG chain degree of polymerization (N_{PEG} = 16, 64), with PLGA chains at fixed length (N_{PLGA} = 64). Snapshots of the four PLGA/PEG mixtures at equilibrium (PEG and PLGA chains are represented as blue and yellow beads, respectively) at atomistic (**C**) and CG (**D**) resolutions.

The miscibility regimes previously characterized at the atomistic level are instead poorly reproduced when using the alternative dimeric P1-C5, Na-N0 and (S)Na-Na configurations (**Figure S4**). In the case of the combination (S)Na-Na, the PLGA-PEG miscibility trend was not reproduced as a likely consequence of the recently emerged undesired effects rising when mixing particles of different sizes described elsewhere.[5]

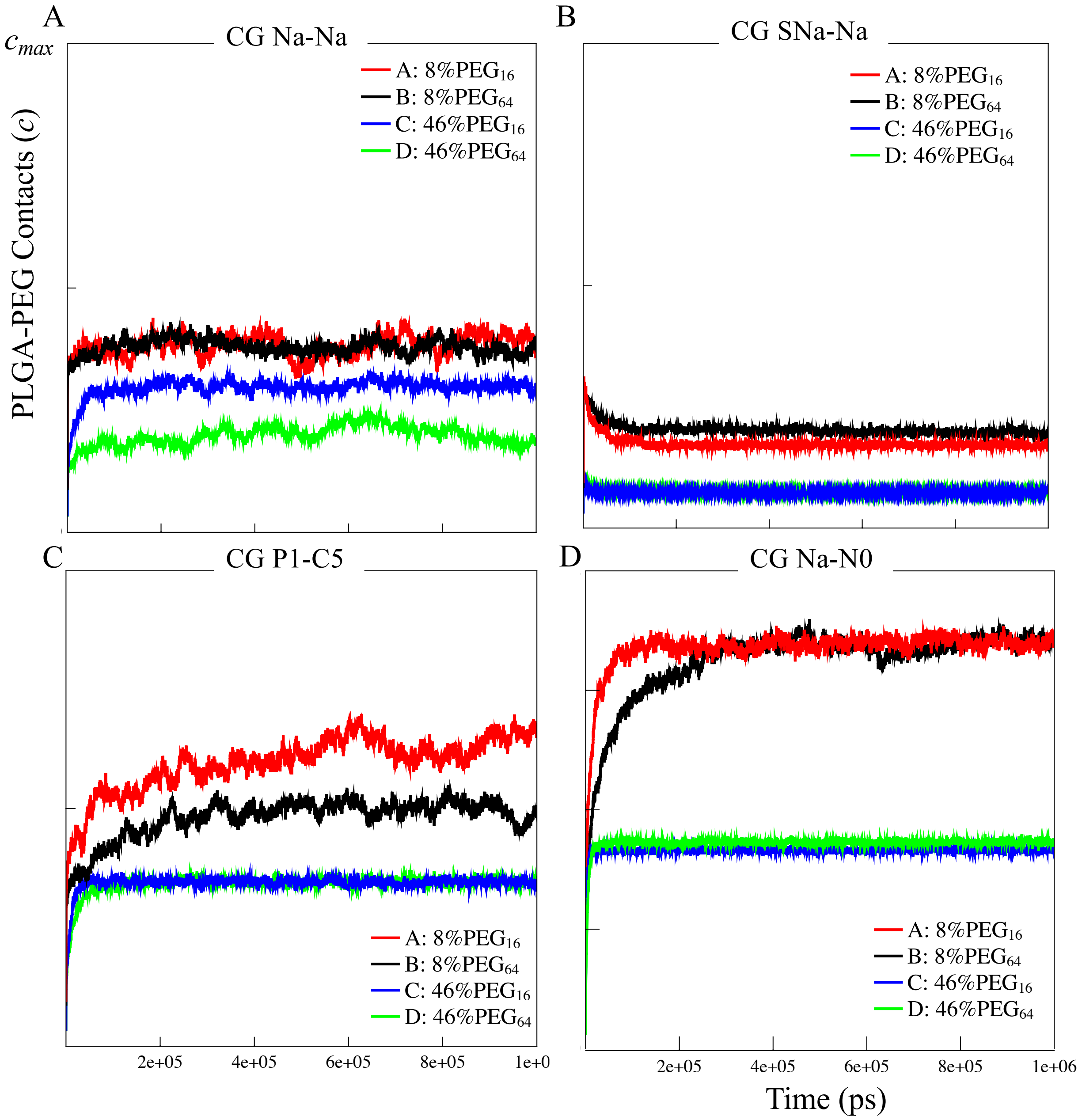

Figure S4. **PLGA/PEG miscibility at coarse-grained resolution (4 mapping schemes).** The normalized number of PLGA/PEG contacts computed over 1 μs is reported as function of the PEG:PLGA ratio (Φ_{PEG} = 8%, 46%) and PEG chain degree of polymerization (N_{PEG} = 16, 64), with

PLGA chains at fixed length (N_{PLGA} = 16) built at coarse-grained resolution from 4 mapping schemes, (**A**) Na-Na, (**B**) SNa-Na, (**C**) P1-C5, and (**D**) Na-N0.

S4. ADDITIONAL SUPPLEMENTARY FIGURES

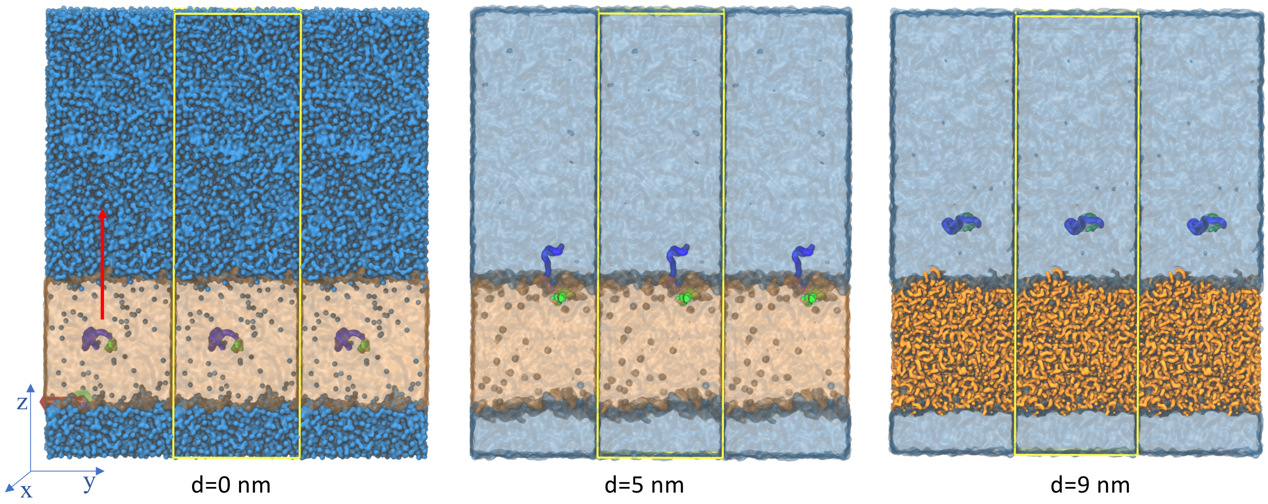

Figure S5. **Setup for umbrella sampling simulations.** From left to right, snapshots of three umbrella windows where the distance between the COM of the drug (PEG25_DTXL, whit PEG chains in dark blue and DTXL in green) and PLGA (orange) is d = 0, 5, and 9 nm, respectively. Water is represented in full or transparent blue.

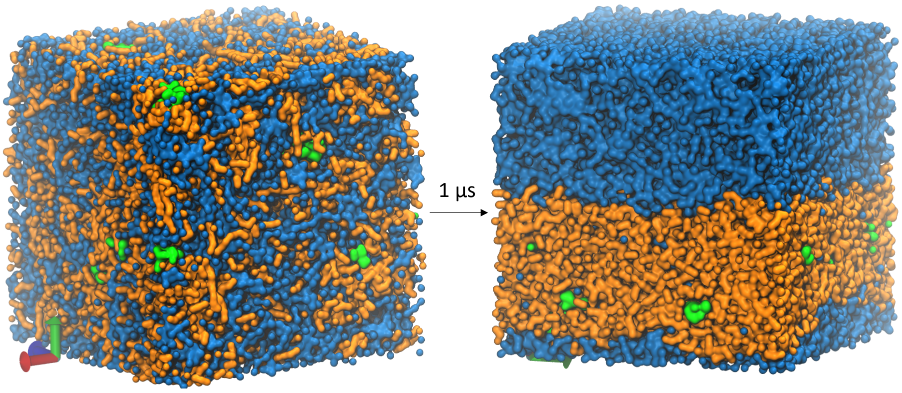

Figure S6. Setup for aggregation propensity. On the left, a snapshot of the initial configuration of the system where PLGA chains (in orange), water molecules (in blue) and DTXL molecules (in green)

are randomly mixed. On the right, the final snapshot of the system after 1 μs of simulation showing the separation between the water and PLGA phases and the DTXL molecules.

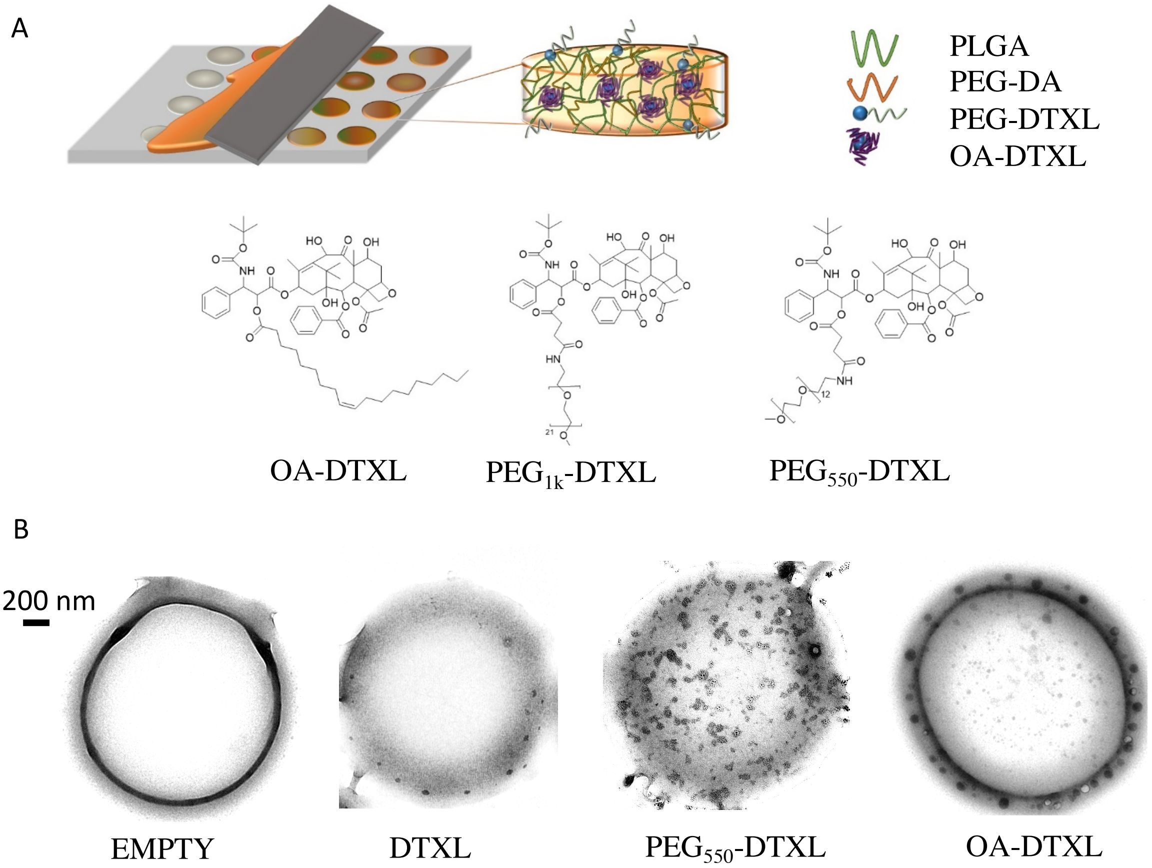

Figure S7. DPN loaded with docetaxel-conjugates. A. Graphical representation of the "direct loading" technique; DPN composition; and structures of the docetaxel-conjugates. **B**. Transmission Electron Microscopy of DPN without any drug molecule (empty); DPN directly loaded with docetaxel (DTXL), with PEG conjugates of docetaxel (PEG_{550}-DTXL), and oleic acid conjugates of docetaxel (DTXL-OA).

A

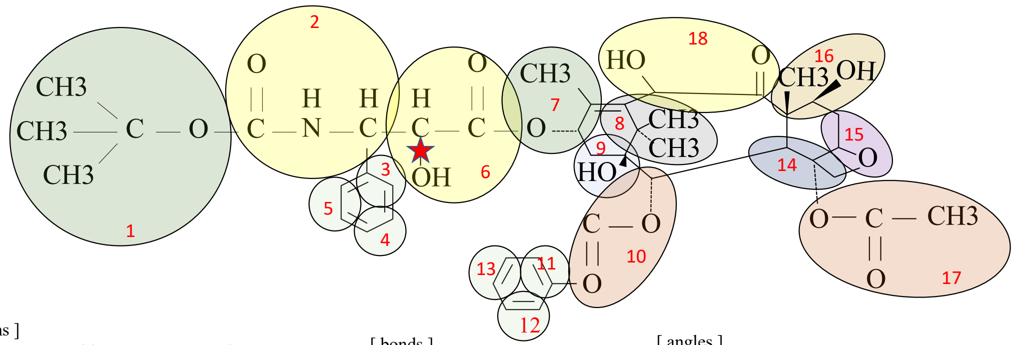

B

[atoms]

; id	type	resnr	residue	atom	cgnr	charge	mass
1	N0	1	DTX	D1	1	0.00000	72.00
2	P3	1	DTX	D2	2	0.00000	72.00
3	SN0	1	DTX	D3	3	0.00000	45.00
4	SN0	1	DTX	D4	4	0.00000	45.00
5	SN0	1	DTX	D5	5	0.00000	45.00
6	P3	1	DTX	D6	6	0.00000	72.00
7	N0	1	DTX	D7	7	0.00000	72.00
8	SC4	1	DTX	D8	8	0.00000	45.00
9	P2	1	DTX	D9	9	0.00000	72.00
10	P1	1	DTX	D10	10	0.00000	72.00
11	SN0	1	DTX	D11	11	0.00000	45.00
12	SN0	1	DTX	D12	12	0.00000	45.00
13	SN0	1	DTX	D13	13	0.00000	45.00
14	SC5	1	DTX	D14	14	0.00000	45.00
15	Sna	1	DTX	D15	15	0.00000	45.00
16	P1	1	DTX	D16	16	0.00000	72.00
17	P1	1	DTX	D17	17	0.00000	72.00
18	P3	1	DTX	D18	18	0.00000	72.00

[bonds]

1	2	1	0.36433	33861.09102
2	3	1	0.38583	14148.52286
2	6	1	0.28874	8433.65018
6	7	1	0.34258	5284.59460
7	9	1	0.34895	44263.94489
9	10	1	0.24121	12837.16752
10	14	1	0.28764	26091.32220
8	9	1	0.24781	20087.58432
8	18	1	0.28878	25113.48530
18	16	1	0.29460	17738.95661
16	15	1	0.30360	39445.30149
14	16	1	0.31624	89940.75416

[constraints]

3	4	1	0.13928
3	5	1	0.20728
4	5	1	0.28911
7	8	1	0.31623
10	11	1	0.32671
11	12	1	0.25748
12	13	1	0.28899
13	11	1	0.29990
15	14	1	0.20943
14	17	1	0.29871

[angles]

1	2	6	2	136.72074	105.25874
1	2	3	2	127.63251	84.25387
6	7	8	2	156.11194	576.14923
6	7	9	2	117.19203	93.32955
7	8	18	2	89.90407	1822.09845
7	9	10	2	125.57804	1217.49531
8	18	16	2	113.39436	1857.35467
8	9	10	2	104.58389	349.74280
9	10	14	2	101.46489	699.35476
9	10	11	2	116.13016	191.09546
10	14	15	2	164.56820	7632.80603
10	14	17	2	111.97131	460.20637
18	16	15	2	139.68438	1123.37426
18	16	14	2	106.94598	947.86882

[dihedrals]

14	17	15	16	1	118.58486.	206.22699	1
17	16	18	8	1	-142.39315	103.31630	1
7	9	18	8	1	-97.05456	533.96090	1
9	10	8	7	1	125.24869	494.63661	1
7	6	9	8	1	-167.01405	165.17089	1
7	18	16	14	1	138.67602	208.19638	1
7	8	18	16	1	105.06501	182.89732	1

C

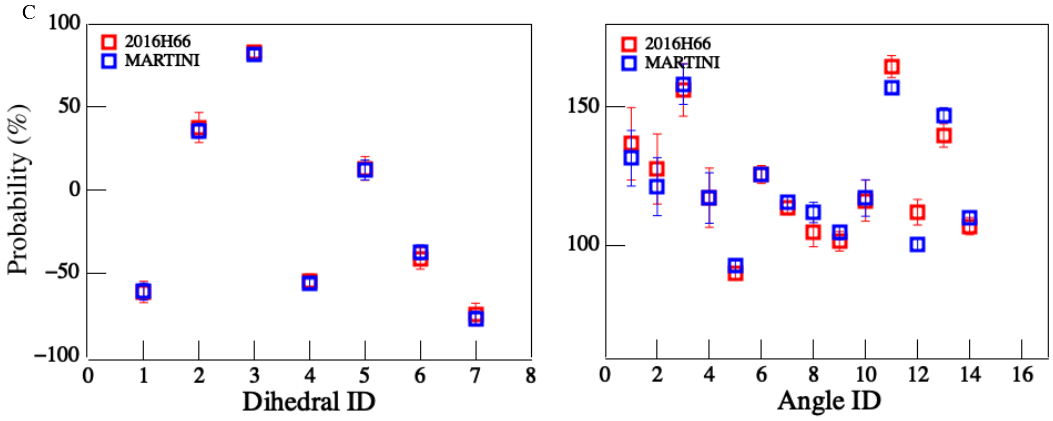

Figure S8. Coarse-grained mapping of Docetaxel. A. CG beads overlap the chemical structure of the target docetaxel molecule. **B**. Bonds, angles, and dihedrals parameters between CG beads defined according to the definition of the MARTINI forcefield and optimized via pycgtool. **C**. Angle and dihedral bonds distributions at atomistic (red line) and coarse-grained resolution (blue line).

SUPPORTING BIBLIOGRAPHY

(1) Flory, P. J. *Principles of polymer chemistry*; Cornell university press, 1953.

(2) Murcia Valderrama, M. A.; van Putten, R.-J.; Gruter, G.-J. M. PLGA Barrier Materials from CO2. The influence of Lactide Co-monomer on Glycolic Acid Polyesters. *ACS applied polymer materials* **2020**, *2* (7), 2706-2718.

(3) Pannuzzo, M.; Horta, B. A. C.; La Rosa, C.; Decuzzi, P. Predicting the Miscibility and Rigidity of Poly(lactic-co-glycolic acid)/Polyethylene Glycol Blends via Molecular Dynamics Simulations. *Macromolecules* **2020**, *53* (10), 3643-3654. DOI: 10.1021/acs.macromol.0c00110.

(4) Pannuzzo, M.; Horta, B. A.; La Rosa, C.; Decuzzi, P. Predicting the Miscibility and Rigidity of Poly (lactic-co-glycolic acid)/Polyethylene Glycol Blends via Molecular Dynamics Simulations. *Macromolecules* **2020**.

(5) Alessandri, R.; Souza, P. C.; Thallmair, S.; Melo, M. N.; De Vries, A. H.; Marrink, S. J. Pitfalls of the Martini model. *Journal of chemical theory and computation* **2019**, *15* (10), 5448-5460.
